# A Rare Constellation of Omphalocele, Myelomeningocele, and Encephalocele in a Neonate Born to a Mother With Type 2 Diabetes Mellitus: A Case Report

**DOI:** 10.1002/ccr3.73872

**Published:** 2026-11-02

**Authors:** Jaber Hamad Jaber Amin, Nabiah Shakeel, Amjed Siddig Khalid Mohammed, Rayan Mohammed, Ahmed Moawia Basher Omer, Amna Magzoob Mahgoob

**Affiliations:** ^1^ University of Sinnar Sinnar Sudan; ^2^ Jinnah Sindh Medical University Karachi Pakistan; ^3^ Faculty of Medicine Alzaiem Alazhari University Khartoum Sudan; ^4^ Al‐Neelain University Khartoum Sudan; ^5^ Nile Valley University Atbara Sudan

**Keywords:** encephalocele: maternal Type 2 diabetes mellitus, myelomeningocele, neural tube defects, omphalocele

## Abstract

Pre‐gestational diabetes with reported poor glycemic control may be associated with rare and severe multiple congenital anomalies, such as omphalocele and multiple concurrent neural tube defects (NTDs). Early antenatal screening, strict maternal glycemic control, and multidisciplinary neonatal management are crucial to improving outcomes.

## 1 Introduction

Major structural anomalies seen in the perinatal period include congenital anomalies of the anterior abdominal wall and of the neural tube and arise during early embryogenesis, particularly during neurulation and ventral body wall formation [1, 2]. These anomalies can happen alone or as part of a multi‐system congenital involvement, which is the result of disruption of early developmental processes [2].

Omphalocele is a defect of the abdominal wall that presents as herniation of abdominal contents through a membranous sac at the base of the umbilical cord [3]. This results from the failure of the midgut to return to the abdominal cavity after physiological herniation [3]. Epidemiological studies revealed that omphalocele is often associated with other congenital anomalies, for example, cardiovascular, gastrointestinal, and central nervous system defects [3, 4].

Neural tube defects are caused by failure of the neural tube to completely close during early embryological development and include myelomeningocele and encephalocele [1]. These conditions are often associated with intracranial abnormalities, including hydrocephalus and Chiari II malformation, which are related to alterations in cerebrospinal fluid dynamics and hindbrain development [5].

Maternal pregestational diabetes mellitus is a well‐established risk factor for a wide range of congenital abnormalities [6]. Population‐based studies have demonstrated a significant association between pregestational diabetes and neural tube defects, as well as other structural malformations, including abdominal wall defects [7]. The teratogenic effects are thought to be mediated, in part, by hyperglycemia‐induced disruption of embryonic signaling pathways during organogenesis [6].

Omphalocele and neural tube defects are individually well characterized; however, their simultaneous occurrence as a “triple” constellation of encephalocele, myelomeningocele, and omphalocele represents a rare and severe pattern of congenital malformation. This case highlights the clinical significance of this unusual combination in the setting of pregestational diabetes mellitus. Our work is in line with the TITAN Guidelines on transparency in AI use in healthcare [8].

## 2 Case History/Examination

A female newborn was delivered at 38 weeks of gestation by emergency cesarean section due to polyhydramnios and fetal distress. The mother was a 35‐year‐old multiparous woman (gravida 10, para 6 + 4) with an eight‐year history of Type 2 diabetes mellitus. She reported adhering to folate supplementation during the periconceptional period, with no history of teratogenic medication exposures, radiation, or illicit substance use. There was no family history of congenital malformations or genetic disorders. The antenatal imaging at 24 weeks of gestation suggested a midline abdominal wall defect and an occipital protrusion. The amniotic fluid volume was significantly increased (1900 mL), although fetal movements remained normal.

The neonate weighed 3.2 kg at birth, with Apgar scores of 7 and 9 at 1 and 5 min, respectively. The head circumference was 38 cm (> 97th percentile). Clinical examination revealed dysmorphic facial features, including a flattened nasal bridge and low‐set ears. A large omphalocele containing the liver and bowel loops was present (Figure 1), along with a lumbosacral cystic lesion measuring 3.2 × 1.8 cm with an exposed neural placode, consistent with myelomeningocele, and an occipital encephalocele (Figures 2, 3, 4). The neonate was hemodynamically stable but exhibited decreased spontaneous movement of the lower extremities. The baseline blood glucose level was 50 mg/dL.

**FIGURE 1:**
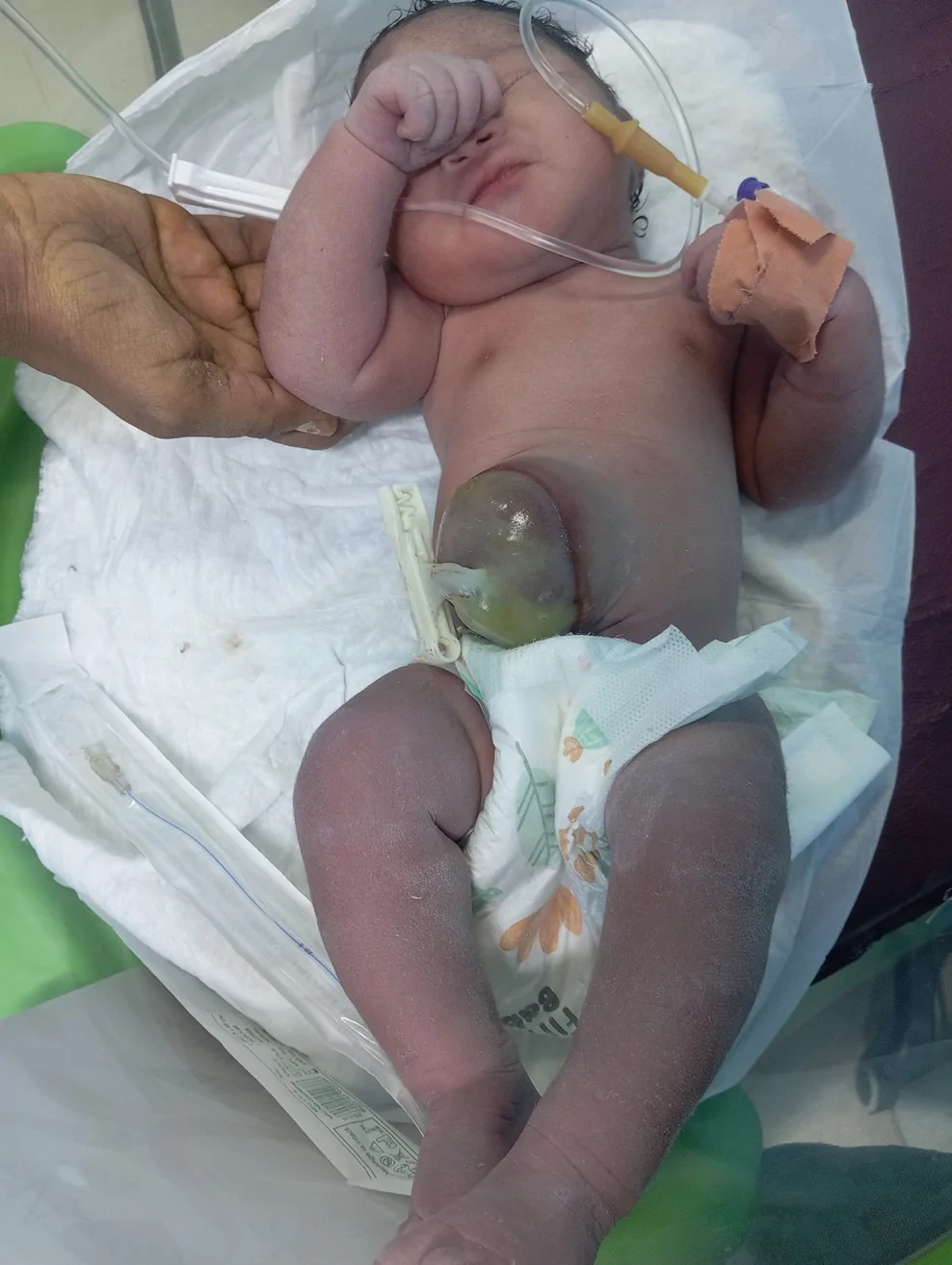
Large omphalocele containing herniated liver and bowel loops within a translucent peritoneal sac at the base of the umbilical cord.

**FIGURE 2:**
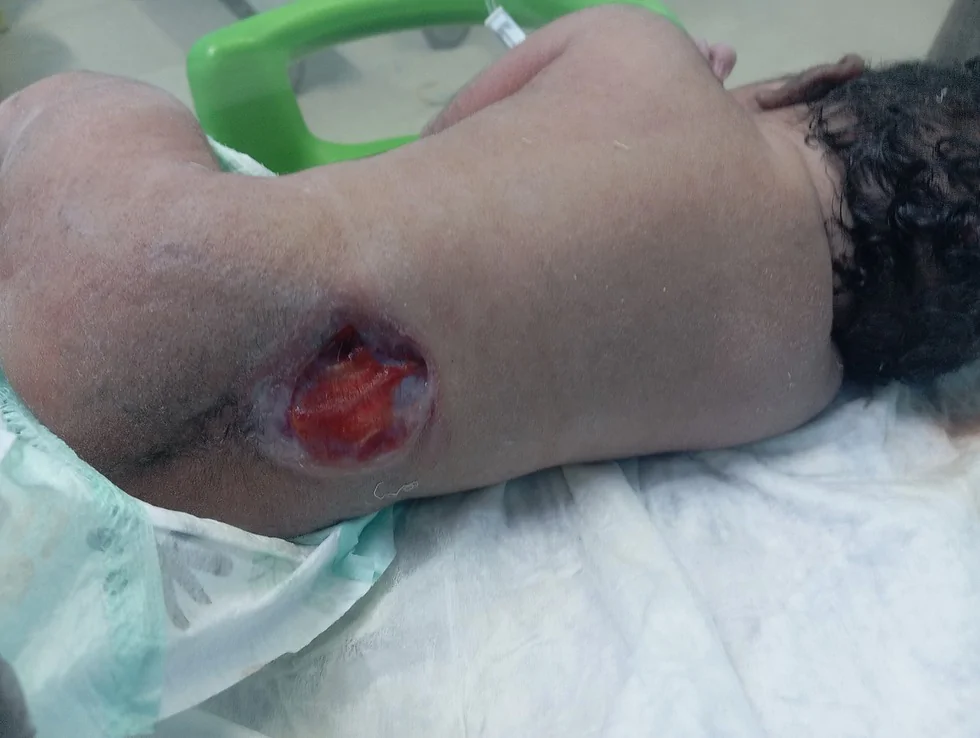
Posterior view showing a lumbosacral myelomeningocele with an exposed neural placode and meningeal membrane before surgical closure.

**FIGURE 3:**
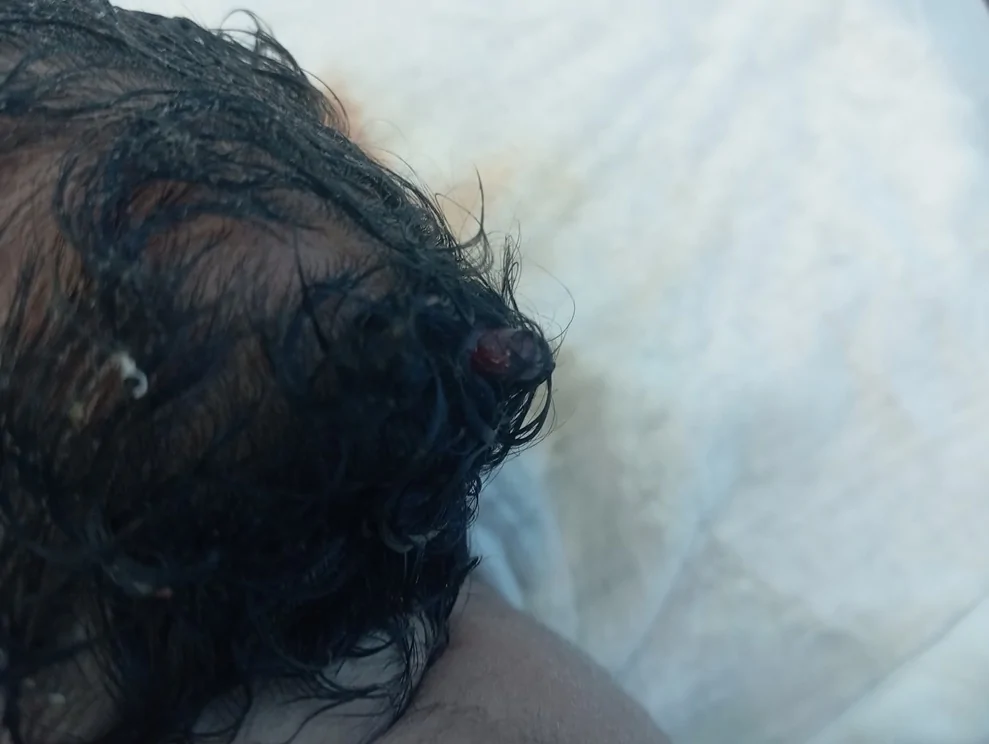
Midline occipital encephalocele presenting as a cystic protrusion through a posterior cranial defect, shown from cranial and caudal views.

**FIGURE 4:**
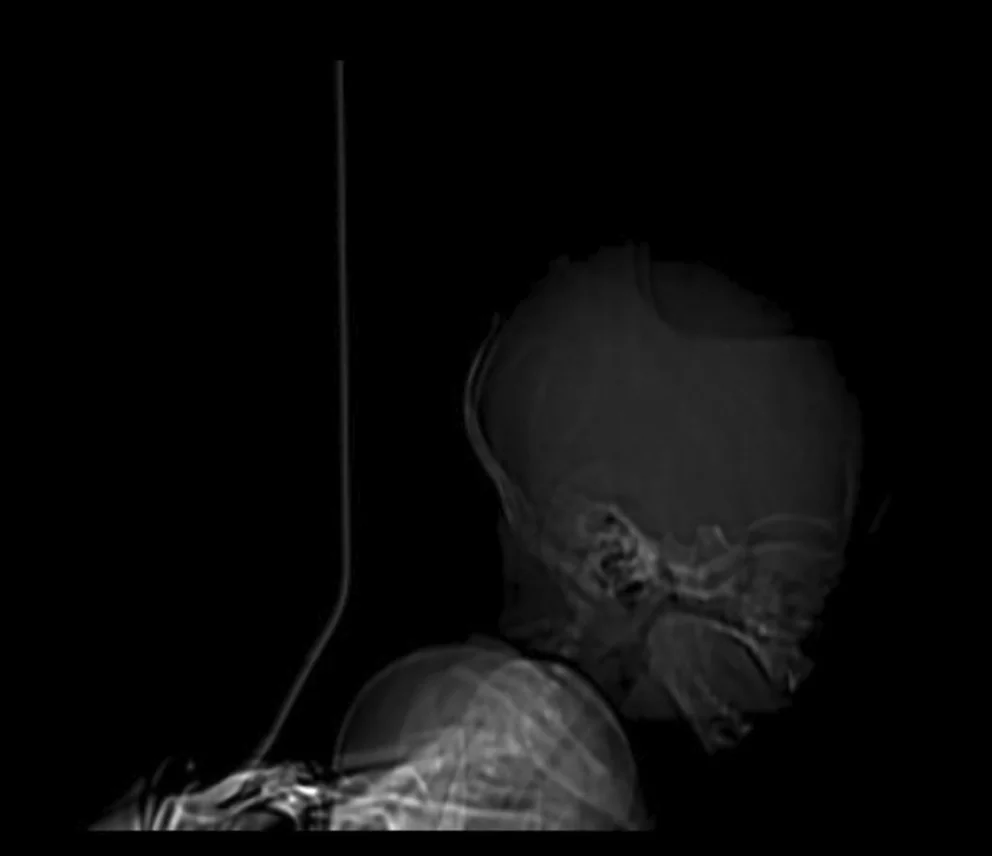
Lateral view of the neonate showing both the occipital encephalocele and lumbosacral myelomeningocele.

## 3 Differential Diagnosis, Investigations and Treatment

Abdominal ultrasonography revealed herniation of the liver and bowel into the omphalocele, while the remaining intra‐abdominal organs appeared normal. Spinal ultrasonography revealed a cystic lumbosacral lesion communicating with the spinal canal, confirming myelomeningocele. Echocardiography demonstrated structurally normal cardiac anatomy. Brain CT and MRI demonstrated obstructive hydrocephalus, with obliteration of the fourth ventricle and asymmetric dilation of the lateral ventricles, together with absence of the posterior arch of C1 and cerebellar tonsillar herniation through the foramen magnum, consistent with Chiari II malformation. No midline shifting or intracranial hemorrhage was observed (Figure 5). The occipital encephalocele contained predominantly meninges and a small portion of gliotic brain tissue. Because the regional resource limited, formal genetic evaluation, karyotyping, or chromosomal microarray testing was not available; so the underlying chromosomal abnormalities or syndromic conditions could not be definitively ruled out.

**FIGURE 5:**
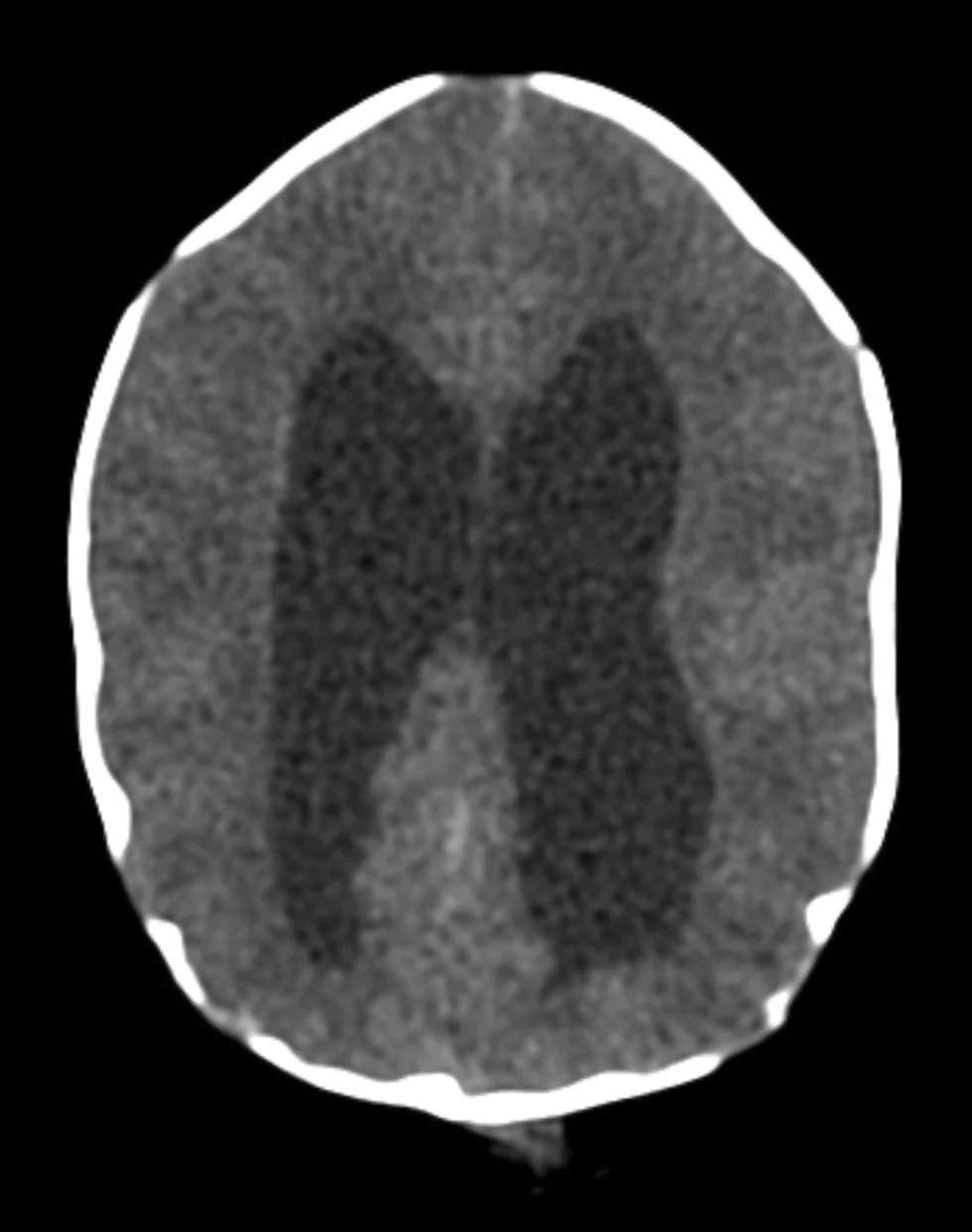
Axial brain computed tomography (CT) neuroimaging demonstrating obstructive hydrocephalus with effacement of the fourth ventricle, consistent with Chiari II malformation.

## 4 Management and Outcome

Initial management included intravenous fluids, empirical antibiotics, vitamin K, glycemic control, and protection of the exposed defects, using sterile saline‐soaked dressings. Early enteral feeding was initiated. Neurosurgical closure of the lumbosacral myelomeningocele was performed on the second day of life, including meticulous dissection, neural placode reconstitution, watertight dural repair, and layered soft‐tissue closure. Hemostasis was achieved, and the postoperative course was uncomplicated. The omphalocele was managed conservatively with topical silver sulfadiazine to promote epithelialization using the “paint and wait” technique. Definitive surgical repair was postponed until clinical stabilization and adequate growth of the abdominal cavity. Regarding the longer‐term care plan, management of progressive hydrocephalus and encephalocele, as well as definitive surgical repair of the omphalocele, remained under ongoing multidisciplinary evaluation.

## 5 Outcome and Follow‐Up

At two‐week follow‐up, the neonate was clinically stable with a well‐healed spinal incision. Neurological assessment showed persistent mild weakness in the lower limbs, but the infant was feeding well and gaining weight.

## 6 Conclusion

The Constellation of Omphalocele, Myelomeningocele, and Encephalocele is an exceedingly rare malformation complex. Although it occurred in the context of maternal Type 2 diabetes, the definitive causation cannot be established given the lack of quantitative glycemic logs (HbA1c) and genetic testing.

## 7 Discussion

Infants born to mothers with diabetes have an increased risk of congenital anomalies and other complications compared with infants born to mothers without diabetes [9]. These anomalies include central nervous system defects such as neural tube defects, hydrocephalus, microcephaly, and caudal regression syndrome [9]. The risk is particularly increased in pregnancies complicated by pregestational diabetes, especially when glycemic control is poor [10]. Chronic maternal hyperglycemia may contribute to diabetic embryopathy through mechanisms involving oxidative stress and the formation of advanced glycation end products [11]. These mechanisms are hypothesized to have potentially contributed to the congenital anomalies observed in this case, given the mother's longstanding Type 2 diabetes mellitus with reported poor glycemic control.

Myelomeningocele and encephalocele are neural tube defects resulting from failure of normal neural tube closure [12]. Myelomeningocele is associated with substantial neurological morbidity, including motor impairment and potential neurodevelopmental difficulties [12]. In the present case, the neonate demonstrated decreased spontaneous movement of the lower extremities at birth and persistent mild lower‐limb weakness at 2‐week follow‐up.

Omphalocele is an uncommon congenital abdominal wall defect that is frequently associated with additional structural anomalies, particularly cardiac, musculoskeletal, gastrointestinal, and central nervous system abnormalities [4, 13]. Advanced maternal age has also been reported among factors associated with omphalocele [14], which is relevant given that the mother in this case was 35 years old.

The distinctive feature of this case is the simultaneous occurrence of three major midline congenital defects: occipital encephalocele, lumbosacral myelomeningocele, and omphalocele. Although maternal diabetes has been independently associated with neural tube defects and abdominal wall defects, the coexistence of these three major anomalies in a single neonate is exceptionally unusual. This constellation highlights the potential severity of complex congenital disruptions and reinforces the importance of optimal glycemic control before conception and throughout pregnancy, together with early antenatal screening and multidisciplinary management.

## 8 Limitation

Despite the clinical educational value of this unusual combination of congenital anomalies, several limitations must be acknowledged. First, quantitative periconceptional records, precise maternal HbA1c levels, serial blood glucose logs, and specific treatment regimens were unavailable. Second, due to resource limitations in our country, it was impossible to conduct any type of genetic testing, including karyotyping and chromosomal microarrays, so the underlying etiologies cannot be entirely excluded. Finally, the scope of this report is limited to acute presentation, initial stabilization, and short‐term outcomes; extended longitudinal data (e.g., the serial head circumference progression, neurodevelopmental trajectories, long‐term urological and bowel function, and the final outcomes of scheduled staged surgeries) are currently unavailable.

## Author Contributions

**Jaber Hamad Jaber Amin:** writing – review and editing, writing – original draft. **Nabiah Shakeel:** writing – original draft, writing – review and editing. **Amjed Siddig Khalid Mohammed:** writing – review and editing, writing – original draft. **Rayan Mohammed:** writing – original draft, writing – review and editing. **Ahmed Moawia Basher Omer:** writing – review and editing, writing – original draft. **Amna Magzoob Mahgoob:** writing – original draft, writing – review and editing.

## Funding

The authors have nothing to report.

## Ethics Statement

As a single‐case report with the parental signed consent, no other ethics review was required.

## Consent

Written informed consent was obtained from the parents for the publication of this case report, with clinical details and identifiable images.

## Conflicts of Interest

The authors declare no conflicts of interest.

## Data Availability

The data that support the findings of this study are available on request from the corresponding author. The data are not publicly available due to privacy or ethical restrictions.

## References

[1] A. J. Copp , N. S. Adzick , L. S. Chitty , J. M. Fletcher , G. N. Holmbeck , and G. M. Shaw , “Spina Bifida,” Nature Reviews. Disease Primers 1 (2015): 15007, 10.1038/nrdp.2015.7. PMC4898641 27189655

[2] T. W. Sadler , “Embryology of Neural Tube Development,” American Journal of Medical Genetics. Part C, Seminars in Medical Genetics 135C, no. 1 (2005): 2–8, 10.1002/ajmg.c.30049. 15806586

[3] C. Stoll , Y. Alembik , B. Dott , and M. P. Roth , “Omphalocele and Gastroschisis and Associated Malformations,” American Journal of Medical Genetics. Part A 146A, no. 10 (2008): 1280–1285, 10.1002/ajmg.a.32297. 18386803

[4] J. Marshall , J. L. Salemi , J. P. Tanner , et al., “Prevalence, Correlates, and Outcomes of Omphalocele in the United States, 1995–2005,” Obstetrics and Gynecology 126, no. 2 (2015): 284–293, 10.1097/AOG.0000000000000920. 26241416

[5] L. E. Mitchell , “Epidemiology of Neural Tube Defects,” American Journal of Medical Genetics. Part C, Seminars in Medical Genetics 135C, no. 1 (2005): 88–94, 10.1002/ajmg.c.30057. 15800877

[6] A. Correa , S. M. Gilboa , L. M. Besser , et al., “Diabetes Mellitus and Birth Defects,” American Journal of Obstetrics and Gynecology 199, no. 3 (2008): 237, 10.1016/j.ajog.2008.06.028. PMC4916956 18674752

[7] K. J. Stothard , P. W. Tennant , R. Bell , and J. Rankin , “Maternal Overweight and Obesity and the Risk of Congenital Anomalies: A Systematic Review and Meta‐Analysis,” Journal of the American Medical Association 301, no. 6 (2009): 636–650, 10.1001/jama.2009.113. 19211471

[8] R. A. Agha , G. Mathew , R. Rashid , et al., “Transparency in the Reporting of Artificial Intelligence–the TITAN Guideline,” Premier Journal of Science 10 (2025): 100082, 10.70389/pjs.100082.

[9] V. Mishra , N. Panigrahi , A. Rao , A. Maheshwari , and T. A. G. M. Huisman , “Neurological Abnormalities in Infants of Mothers With Diabetes Mellitus,” Journal of Neonatology 36, no. 2 (2022): 150–158, 10.5005/jp-journals-11002-0056.

[10] Y. Wu , B. Liu , Y. Sun , et al., “Association of Maternal Prepregnancy Diabetes and Gestational Diabetes Mellitus With Congenital Anomalies of the Newborn,” Diabetes Care 43, no. 12 (2020): 2983–2990, 10.2337/dc20-0261. PMC7770264 33087319

[11] U. J. Eriksson and P. Wentzel , “The Status of Diabetic Embryopathy,” Upsala Journal of Medical Sciences 121, no. 2 (2016): 96–112, 10.3109/03009734.2016.1165317. PMC4900070 27117607

[12] M. Akar , G. Çetin , M. İnal , et al., “Postnatal Surgery for Myelomeningocele in Neonates: Neurodevelopmental Outcomes,” Turkish Journal of Medical Sciences 53, no. 1 (2023): 88–93, 10.55730/1300-0144.5561. PMC10388020 36945937

[13] M. S. V. Pereira , D. K. R. Vieira , M. F. M. P. Leite , M. A. M. Villar , and C. V. B. Farias , “Omphalocele: Clinical and Epidemiological Profile of Patients Born in a Tertiary Care Center in Rio de Janeiro,” BMC Pregnancy and Childbirth 23, no. 1 (2023): 424, 10.1186/s12884-023-05741-z. PMC10245419 37286958

[14] C. Stoll , Y. Alembik , B. Dott , and M. P. Roth , “Risk Factors in Congenital Abdominal Wall Defects (Omphalocele and Gastroschisi): A Study in a Series of 265,858 Consecutive Births,” Annales de Génétique 44, no. 4 (2001): 201–208, 10.1016/S0003-3995(01)01094-2. 11755106

